# Plasma Thallium Concentration, Kidney Function, Nephrotoxicity and Graft Failure in Kidney Transplant Recipients

**DOI:** 10.3390/jcm11071970

**Published:** 2022-04-01

**Authors:** Daan Kremer, Niels L. Riemersma, Dion Groothof, Camilo G. Sotomayor, Michele F. Eisenga, Adrian Post, Tim J. Knobbe, Daan J. Touw, Stephan J. L. Bakker

**Affiliations:** 1 Department of Internal Medicine, Division of Nephrology, University of Groningen and University Medical Center Groningen, 9700 RB Groningen, The Netherlands; n.l.riemersma@umcg.nl (N.L.R.); d.groothof@umcg.nl (D.G.); c.g.sotomayor.campos@umcg.nl (C.G.S.); m.f.eisenga@umcg.nl (M.F.E.); a.post01@umcg.nl (A.P.); t.j.knobbe@umcg.nl (T.J.K.); s.j.l.bakker@umcg.nl (S.J.L.B.); 2 Institute of Biomedical Sciences, Faculty of Medicine, University of Chile, Santiago 8380453, Chile; 3 Radiology Department, Clinical Hospital University of Chile, University of Chile, Santiago 8380453, Chile; 4 Department of Clinical Pharmacology, University of Groningen and University Medical Center Groningen, 9700 RB Groningen, The Netherlands; d.j.touw@umcg.nl

**Keywords:** nephrotoxicity, tubular damage, kidney transplantation, graft failure, heavy metals

## Abstract

The nephrotoxic effects of heavy metals have gained increasing scientific attention in the past years. Recent studies suggest that heavy metals, including cadmium, lead, and arsenic, are detrimental to kidney transplant recipients (KTR) even at circulating concentrations within the normal range, posing an increased risk for graft failure. Thallium is another highly toxic heavy metal, yet the potential consequences of the circulating thallium concentrations in KTR are unclear. We measured plasma thallium concentrations in 672 stable KTR enrolled in the prospective TransplantLines Food and Nutrition Biobank and Cohort Study using inductively coupled plasma mass spectrometry. In cross-sectional analyses, plasma thallium concentrations were positively associated with kidney function measures and hemoglobin. We observed no associations of thallium concentration with proteinuria or markers of tubular damage. In prospective analyses, we observed no association of plasma thallium with graft failure and mortality during a median follow-up of 5.4 [interquartile range: 4.8 to 6.1] years. In conclusion, in contrast with other heavy metals such as lead, cadmium, and arsenic, there is no evidence of tubular damage or thallium nephrotoxicity for the range of circulating thallium concentrations observed in this study. This is further evidenced by the absence of associations of plasma thallium with graft failure and mortality in KTR.

## 1. Introduction

Late kidney allograft failure is a constant threat to kidney transplant recipients (KTR), necessitating re-initiation of dialysis and/or re-transplantation [1]. Therefore, the search for potential modifiable causes of graft failure is ongoing. Heavy metal-induced nephrotoxicity may be a potential cause of kidney graft failure that is often overlooked, yet it has gained international attention in the past years [2,3,4,5]. While the nephrotoxic effect of circulating high concentrations of heavy metals is well-established [6], the KTR population may already be vulnerable to nephrotoxicity at concentrations that would otherwise be considered within the normal range. This high vulnerability is likely because KTR generally have only one functioning kidney that is at risk of ischemia-reperfusion injury during the transplantation procedure and potential hyperperfusion after transplantation [7]. Multiple concomitant diseases (e.g., diabetes and cardiovascular disease) and strict adherence to an immunosuppressive maintenance regimen (usually including persistent exposure to nephrotoxic calcineurin inhibitors [8]) further add to this strain. Clearly, these circumstances greatly limit the resilience of the kidney’s ability to handle additional unfavorable factors, such as exposure to heavy metals. Indeed, it was recently found that high-normal circulating concentrations of heavy metals, including cadmium, lead, and arsenic are independently associated with a higher risk of graft failure in KTR [3,4,5].

Thallium, another heavy metal, is more acutely toxic than the aforementioned heavy metals [9]. Humans are generally exposed to thallium via air, water, and food, especially in the vicinity of mining areas, urban-runoff rivers, and factories. However, most exposure occurs via the contamination of food [10,11,12]. Symptoms of acute thallium poisoning include gastrointestinal complaints and neurological symptoms, including polyneuropathy, but the condition can also be fatal [13,14,15]. Chronic thallium toxicity can occur after long-term low-level thallium exposure, generally presenting with anorexia, headaches, and neurological symptoms [10,16]. Thallium exposure is associated with nephrotoxicity in rats [17,18], but studies assessing the potential nephrotoxic effects of thallium in humans are lacking [9].

We therefore aimed to determine plasma thallium concentration and assess the associations of clinical and biochemical parameters with plasma thallium concentration. Additionally, we investigated whether plasma thallium concentration is associated with kidney graft survival and patient survival in a cohort of stable KTR.

## 2. Materials and Methods

### 2.1. Design and Study Population

For this prospective cohort study, we used data from patients enrolled in the TransplantLines Food and Nutrition Biobank and Cohort Study. All KTR (≥18 years old) visiting the University Medical Centre Groningen (UMCG) outpatient clinic with a functioning graft for approximately 1 year or longer after transplantation, and without any known drug or alcohol addiction or systemic illnesses, were invited for participation [19]. In the period between November 2008 and March 2011, 707 KTR were included in the cohort. All KTR were transplanted at the UMCG and were treated with standard antihypertensive and immunosuppressive treatments. Data in this cohort included a broad variety of biochemical parameters, questionnaires, and anthropological measurements. Notably, these analyses were performed on the same population that recent studies had shown evidence of nephrotoxicity due to cadmium, lead, and arsenic [3,4,5]. For the current study, participants without available samples for plasma thallium measurements were excluded. The study was conducted according to the guidelines laid down in the Declaration of Helsinki and the study protocol was approved by the Institutional Review Board (METc 2008/186). The flow of participants through the study is detailed in Supplementary Figure S1.

The primary outcome of this study was death-censored graft failure, defined as end-stage kidney disease requiring dialysis or re-transplantation. A secondary outcome was all-cause mortality. There were no losses to follow-up.

### 2.2. Outcome Measurements

All measurements were performed during a morning visit to the outpatient clinic, as described in detail previously [19]. Blood pressure and heart rate were measured with a semi-automatic device (Dinamap 1846, Critikon, Tampa, FL, USA) every minute for a period of 15 min, and the last three measurements were averaged. Body weight and height were measured with the participants wearing indoor clothing without shoes. Body mass index (BMI) was calculated as weight in kilograms divided by height in meters squared (kg/m^2^). Information on medical history and medication was extracted from patient records. Information on smoking behavior and alcohol intake was obtained by using a questionnaire. Diabetes was based on the definition of the American Diabetes Association. The estimated glomerular filtration rate (eGFR) was calculated using the creatinine- and cystatin C-based CKD-EPI formula [20].

### 2.3. Laboratory Methods and Thallium Measurement

Blood was drawn in the morning after an 8 to 12 h overnight fasting period after the completion of 24 h urine collection. In addition, all participants were instructed to collect 24 h urine during the day before their visit to the outpatient clinic.

Plasma thallium concentrations were measured in ethylenediaminetetraacetic acid (EDTA) plasma samples, which were stored frozen until measurement at −80 °C. Plasma concentrations of thallium, cadmium, lead, and arsenic were determined by inductively coupled plasma mass spectrometry (Varian 820-MS; Varian, Palo Alto, CA, USA) with a modified method for the measurement of low concentrations of heavy metals in plasma using a standard addition method, as described elsewhere [5].

Cystatin C concentration was measured in EDTA plasma using a validated particle-enhanced turbidimetric immunoassay (Gentian, Moss, Norway). Each patient collected 24 h urine prior to the baseline study visit following strict instructions. Total urinary protein excretion was determined by means of the Biuret reaction (MEGA AU 510; Merck Diagnostica). Plasma neutrophil-gelatinase associated lipocalin (NGAL) concentration was measured in EDTA-plasma using a validated particle-enhanced turbidimetric immunoassay (Gentian, Moss, Norway). Urinary liver-type fatty acid-binding protein (L-FABP) was measured with an enzyme-linked immunosorbent assay (human uL-FABP assay kit 96 test; CMIC holdings Co., Tokyo, Japan). Urinary endothelial growth factor (EGF) concentration was measured by ELISA (R&D Systems, Minneapolis, MN, USA) and divided by the urinary creatinine concentration. Other laboratory parameters, including creatinine, high-sensitivity C-reactive protein (hs-CRP), and glycated hemoglobin (HbA1c) were measured using routine laboratory methods.

### 2.4. Statistical Analyses

Baseline characteristics were summarized as the mean ± SD, median [interquartile range], and count (%) for normally distributed, non-normally distributed, and nominal data, respectively. Univariable linear regression analyses were performed to assess associations of plasma thallium concentration with clinical and biochemical parameters. Scatterplots and Q-Q plots were visually evaluated to assess whether the assumptions of linear regression analyses were met. Non-normally distributed variables were log_2_ transformed when necessary to meet these assumptions. Listwise exclusion was applied in the case of missing data; the number of missing values is indicated in table footnotes.

The association of plasma thallium concentration with graft failure and mortality were assessed with Kaplan–Meier curves and the significance of the difference between the tertiles was calculated using a log-rank test. To further assess the association of plasma thallium concentration with graft failure and all-cause mortality, Cox-proportional hazards regression analyses were performed with adjustments for potential confounders, including age, sex, 24 h urinary protein excretion, eGFR, history of cardiovascular disease, pre-emptive transplantation, and plasma concentrations of other heavy metals. There was no evidence for non-proportionality of hazards in any of the Cox models (*p* > 0.05 for all). Potential interaction of thallium with age, sex, or eGFR was assessed by adding interaction terms to the Cox models.

In order to identify whether relatively high plasma thallium concentrations were associated with an impaired outcome, the Kaplan–Meier curves and log-rank tests were repeated to compare graft and patient survival among patients with a plasma thallium concentration >90th and ≤90th percentile. These analyses were repeated with the 95th percentile as a cut-off.

In additional sensitivity analyses, Cox regression analyses were repeated, where the thallium concentrations were transformed using log_2_, the square root, the square, and the inverse of each value. In addition, potential non-linear associations of plasma thallium with outcome were assessed by adding restricted cubic spline terms for thallium to the models. The statistical significance of the improvements in model fit was assessed using likelihood ratio tests.

All data were analyzed using R version 4.0.5 (Vienna, Austria). For all analyses, *p* < 0.05 indicated statistical significance.

## 3. Results

### 3.1. Baseline Characteristics

In total, 672 KTR were included in this study. The mean age was 53 ± 13 years, 42% were female, the median time after transplantation was 5.4 [1.9 to 11.8] years at the time of inclusion, and the mean eGFR was 45 ± 19 mL/min/1.73 m^2^. The mean plasma thallium concentration was 0.12 ± 0.06 µg/L. The baseline characteristics are presented in more detail in Table 1.

### 3.2. Associations of Plasma Thallium with Clinical and Kidney Function Parameters

**Figure 1:**
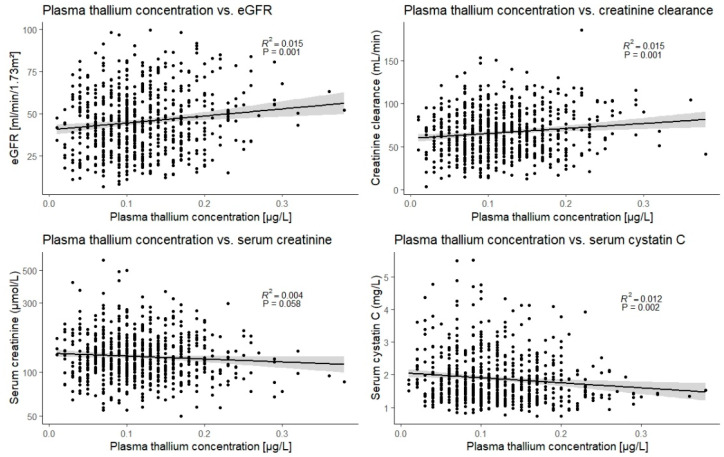
Scatter plots and visual presentation of the univariable linear association of plasma thallium concentration with the parameters of kidney function.

In linear regression analyses, a higher age was associated with a lower plasma thallium concentration (Table 1). In addition, thallium concentration was positively associated with hemoglobin (St.β 0.15; 95% CI: 0.07 to 0.22). Notably, eGFR and creatinine clearance were positively associated with plasma thallium concentration, whereas cystatin C was negatively associated with plasma thallium concentration (Figure 1, *p* < 0.01 for all). The association of plasma thallium with serum creatinine, however, was not statistically significant. Additional explanatory analyses revealed that the associations of thallium with eGFR, creatinine clearance, and serum cystatin C disappeared after an adjustment for hemoglobin (*p* > 0.05 for all, respectively).

Interestingly, we observed a negative association of plasma thallium concentration with plasma cadmium concentration (St.β −0.14; 95% CI: −0.21 to −0.06), but it was not associated with plasma concentrations of lead, arsenic, and mercury.

### 3.3. Associations of Plasma Thallium with Parameters of Nephrotoxicity and Tubular Damage

**Figure 2:**
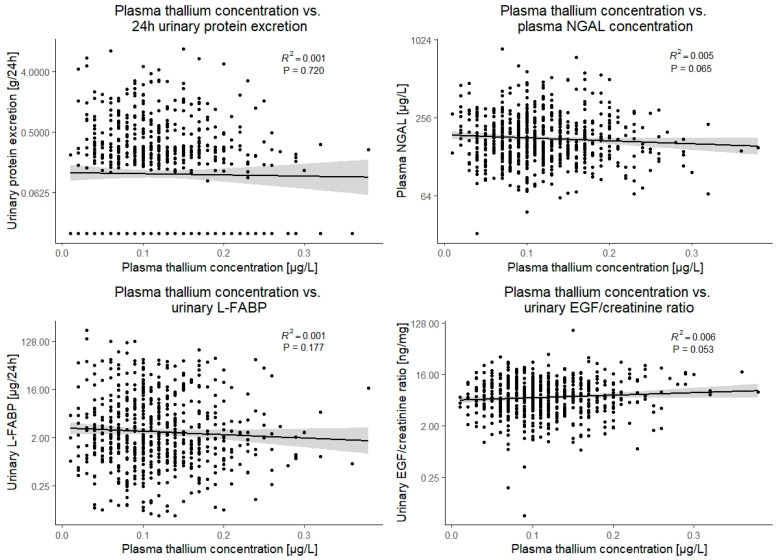
Scatter plots and visual presentation of the linear association of plasma thallium concentration with the parameters of tubular damage. EGF, endothelial growth factor; NGAL, neutrophil-gelatinase associated lipocalin; L-FABP, liver-type fatty acid-binding protein.

Plasma thallium concentration was not associated with any of the markers of tubular damage, including 24 h urinary protein excretion, plasma neutrophil-gelatinase associated lipocalin, urinary liver-type fatty acid binding protein (L-FABP), or the urinary endothelial growth factor (EGF)/creatinine ratio (*p* > 0.05 for all). Notably, although insignificant, point estimates of the association of thallium with urinary EGF/creatinine ratio and plasma neutrophil-gelatinase associated lipocalin were consistent with tubular protection rather than damage (St.β 0.08; 95%CI: −0.13 to 0.03 and St.β −0.07; 95%CI: −0.15 to 0.00, respectively). These associations are visually presented in Figure 2.

### 3.4. Associations with Outcome

**Figure 3:**
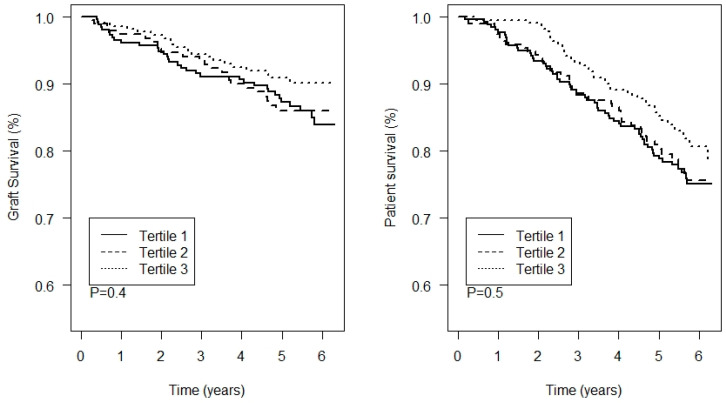
Kaplan–Meier analyses for death-censored graft survival and patient survival per tertile of plasma thallium. The *p*-value represents the significance of difference between the groups as assessed by a log-rank test.

During a median follow-up of 5.3 [IQR: 4.8 to 6.1] years, 80 (11.9%) KTR developed graft failure and 143 (21.3%) died. There were no significant differences between the tertiles of thallium in graft failure and all-cause mortality (*p* = 0.4 and *p* = 0.5, respectively; Figure 3). Notably, plasma thallium concentration tended to be associated with a slightly lower risk of graft failure, although this result did not reach statistical significance. In addition, univariable and multivariable Cox proportional hazards models showed no prospective associations between thallium and all-cause mortality or graft failure, as shown in Table 2. There were no interactions of thallium with age, sex, or eGFR for the association with graft failure or mortality.

### 3.5. Sensitivity Analyses

Patients with a plasma thallium concentration >90th percentile (i.e., >0.19 µg/L, including 67 patients) or >95th percentile (i.e., >0.22 µg/L, including 34 patients) were no more or less at risk of graft failure or mortality compared to the rest of the population (Supplementary Figures S2 and S3). Additional sensitivity analyses where thallium was transformed also showed no significant associations of thallium with outcome (Supplementary Table S1). Finally, models including spline terms for plasma thallium did not improve the model fit for graft failure (*p* = 0.3) or mortality (*p* = 0.9) compared to models without these terms, and none of the spline terms were significantly associated with either outcome (*p* > 0.5 for all).

## 4. Discussion

In this large cohort of 672 well-characterized KTR, thallium was positively associated with eGFR, but this association was no longer present after adjustment for hemoglobin. We also observed no cross-sectional associations of plasma thallium concentration with urinary protein excretion and markers of tubular damage. In addition, plasma thallium concentration was not associated with graft failure or all-cause mortality.

Recent observations have raised major concerns regarding the nephrotoxicity of heavy metals, especially in populations with kidney disease, such as KTR [3,4,5]. Recent studies have shown that circulating plasma concentrations of heavy metals such as lead, cadmium, and arsenic were all independently associated with graft failure, even at concentrations that are generally considered to be within the normal range [3,4,5]. We therefore studied associations of the circulating concentration of another toxic heavy metal, thallium, in the same cohort of KTR as these aforementioned studies.

Previous studies have shown that exposure to thallium can affect human health, but studies of thallium toxicity in low chronic doses is lacking [9]. Natural exposure to thallium can occur through air, water, and food, especially in the vicinity of mining areas, urban-runoff rivers, and factories [10,11,12]. Coal burning power plants, cement factories, and smelting operations pollute the air and rivers with thallium [22]. Subsequently, the heavy metal in the air falls the onto the land, where it is taken up by the crops via their roots and foliage. When this regional low-level exposure is increased, it may become harmful for the population. Symptoms of anorexia, headache, fatigue, polyneuropathic symptoms, pain, and sleep disorders can develop after long-lasting thallium exposure [10,16].

In our study population, mean plasma thallium concentration among KTR was 0.11 µg/L, which is evidently lower than the upper limit of the normal concentration of 2.5 µg/L suggested by the World Health Organization [23], and slightly lower than the mean thallium concentration in a previous study of 350 healthy Italians [24].

After intake, thallium is almost entirely absorbed from the gastro-intestinal tract and quickly deposited in soft tissues, bones, and organs [25]. Accumulation of thallium is particularly high in the kidneys, followed by accumulation in the bones, stomach, intestines, and other organs [26]. Because the kidneys accumulate thallium—and because of the suggested nephrotoxic effects of other heavy metals in this population—we hypothesized that kidney function would be lower in patients with higher plasma thallium concentrations. Our results, on the contrary, suggest a positive association between kidney function and plasma thallium concentration. Notably, this finding is in line with previous studies in the general population, and among lead workers, which showed that higher urinary thallium levels were associated with higher kidney function [27,28,29,30,31]. Although these associations were statistically significant, their clinical relevance appears limited: less than 2% of the variation in kidney function could be explained by variations in thallium concentration. Thallium was also associated with hemoglobin in our study, and we may therefore speculate that circulating thallium concentrations are dependent on hemoglobin, similar to other heavy metals [32,33,34]. Following this logic, and given the established association of kidney function with hemoglobin, we hypothesized that hemoglobin may explain the association of kidney function with the circulating thallium concentration. This notion was indeed supported by our study because the associations of thallium with kidney function disappeared after adjusting for hemoglobin. Nonetheless, these findings consistently suggest that low circulating thallium concentrations are not nephrotoxic, even in populations with kidney disease, such as KTR.

To further assess potential nephrotoxicity, we studied associations of plasma thallium concentration with parameters of tubular damage, including urinary protein excretion, plasma NGAL, urinary L-FABP, and the urinary EGF/creatinine ratio [35,36,37]. Recently, it was shown in the same cohort that plasma cadmium concentration was indeed associated with these parameters, indicating nephrotoxicity [5]. Our current study showed that plasma thallium was not associated with these markers of tubular damage, again suggesting that thallium concentrations in the observed range do not exert nephrotoxic effects.

The lack of nephrotoxic effects of thallium was further corroborated in prospective analyses, where plasma thallium concentrations were not associated with either graft failure or mortality. We may hypothesize that thallium nephrotoxicity may only occur in patients with a relatively high plasma thallium concentration. However, our sensitivity analyses also showed no evidence of the detrimental effects of thallium in patients with plasma thallium concentrations >90th or >95th percentiles. These findings suggest that, in contrast with other heavy metals, including cadmium and lead, circulating thallium concentrations in the range that we investigated are not a major concern for kidney health, which may be relevant for other populations. However, we cannot exclude the possibility that thallium concentrations higher than those observed in our cohort can exert nephrotoxic effects. Whether higher thallium exposures may have nephrotoxic effects therefore remains to be determined in other populations.

The observed negative association of plasma thallium with plasma cadmium concentration may appear surprising. They are in line, however, with observations by Shelley et al., in a study of lead workers, where a similar negative association between urinary thallium and cadmium levels was observed [27]. Although the underlying causes for this association remain unknown, our findings further support the statement by Shelley et al. that multiple metal analysis approaches are needed to gain a better understanding of heavy metal handling and nephrotoxicity [27].

A major strength of this study is the large cohort of well-characterized KTR. This allowed us to assess the associations of plasma thallium with many clinical parameters and the parameters of kidney function and tubular damage. A second strength of this study is that the included patients were followed for a long period without losses to follow-up, which allowed us to thoroughly assess the long-term association between plasma thallium levels and graft failure. Despite the long follow-up, we cannot exclude the possibility that potential detrimental effects of thallium become clear only during a longer follow-up. However, our study population consists mainly of Caucasian participants, included from a single transplantation center in the Netherlands. This calls for prudence when extrapolating these results to other populations and geographical areas with potentially different local thallium exposure. Future studies in different populations from different geographical areas with potentially higher thallium exposure are therefore needed. Such future studies may potentially also investigate thallium inhaled in small particulate matter, as the latter has been shown to be associated with the occurrence of membranous nephropathy [38]. Finally, we only assessed thallium levels in plasma. Several previous studies found that urinary thallium concentration showed similar associations with kidney function as the plasma concentration in our study [27,28,29,30,31]. Future studies on urinary thallium and thallium clearance may be useful for providing more insight on the effects of thallium on the kidney. In addition, determination of thallium in other media, such as nails or hair, may be helpful when exploring the potential effects of long-term thallium exposure in future studies [39,40].

## 5. Conclusions

Circulating thallium concentrations are not associated with markers of nephrotoxicity, or with graft failure or mortality in KTR. In contrast with other heavy metals such as lead, cadmium, and arsenic, there is no evidence of tubular damage or thallium nephrotoxicity for the range of circulating thallium concentrations observed in this study.

## Figures and Tables

**Table 1:** Population characteristics at baseline and linear regression analyses for plasma thallium concentration.

	N = 672	Linear Regression with Plasma Thallium as Dependent Variable	
Plasma thallium (µg/L)	0.12 (0.06)	St. β (95% CI)	*p*-Value
**Clinical characteristics**			
Female sex, *n* (%)	285 (42%)	−0.08 (−0.23 to 0.07)	0.3
Age, y	53 (13)	−0.12 (−0.20 to −0.04)	0.002
Primary renal disease, *n* (%)			
Unknown	103 (15%)	Ref.	
Glomerulonephritis	176 (26%)	0.08 (−0.17 to 0.32)	0.5
Interstitial nephritis	82 (12%)	0.12 (−0.18 to 0.41)	0.4
Cystic kidney disease	139 (20%)	0.02 (−0.27 to 0.24)	0.9
Other congenital/hereditary disease	39 (6%)	0.01 (−0.38 to 0.36)	0.9
Renal vascular disease	37 (6%)	−0.09 (−0.47 to 0.29)	0.6
Diabetes mellitus	32 (5%)	−0.14 (−0.54 to 0.29)	0.5
Other multisystem diseases	45 (7%)	0.06 (−0.30 to 0.41)	0.8
Other	19 (3%)	−0.01 (−0.48 to 0.51)	0.9
Height, cm	174 (10)	0.02 (−0.06 to 0.09)	0.7
Weight, kg	80.7 (16.5)	0.02 (−0.06 to 0.09)	0.6
Body surface area, m^2^	1.95 (0.22)	0.02 (−0.06 to 0.10)	0.6
Body mass index, kg/m^2^	26.7 (4.8)	0.01 (−0.06 to 0.09)	0.8
Systolic blood pressure, mmHg	136 (17)	−0.07 (−0.15 to 0.01)	0.07
Diabetes, *n* (%)	161 (24%)	−0.01 (−0.19 to 0.17)	0.9
History of cardiovascular disease, *n* (%)	162 (24%)	0.00 (−0.18 to 0.18)	0.9
Smoking status, *n* (%)			
Never	267 (42%)	Ref.	
History of smoking	285 (45%)	−0.02 (−0.19 to 0.15)	0.8
Current smoking	80 (13%)	0.11 (−0.15 to 0.36)	0.4
Pre-emptive transplantation, *n* (%)	107 (16%)	−0.06 (−0.27 to 0.15)	0.6
Duration of dialysis, months ^#^	23 [5 to 47]	0.02 (−0.05 to 0.10)	0.6
Time after transplantation, y ^#^	5.4 [1.9 to 12.8]	0.03 (−0.05 to 0.10)	0.5
History of rejection, *n* (%)	177 (26%)	0.05 (−0.12 to 0.22)	0.6
History of delayed graft function, *n* (%)	50 (7%)	−0.06 (−0.35 to 0.23)	0.7
Living donor, *n* (%)	232 (35%)	0.04 (−0.12 to 0.20)	0.6
**Routine laboratory measurements**			
Sodium mmol/L	140.9 (2.8)	0.00 (−0.08 to 0.08)	0.9
Potassium, mmol/L	4.0 (0.5)	−0.03 (−0.05 to 0.10)	0.5
HbA1c, % ^#^	5.8 [5.5 to 6.2]	0.02 (−0.06 to 0.10)	0.6
Hemoglobin, mmol/L	8.2 (1.1)	0.15 (0.07 to 0.22)	<0.001
Leukocyte count, 10^9^/L	8.1 (2.6)	0.05 (−0.02 to 0.13)	0.2
hs-CRP, mg/L ^#^	1.6 [0.7 to 4.6]	−0.01 (−0.08 to 0.07)	0.9
**Kidney function parameters**			
Cystatin C, mg/L	1.88 (0.77)	−0.12 (−0.19 to −0.04)	0.002
Creatinine, µmol/L ^#^	124 [99 to 159]	−0.07 (−0.15 to 0.00)	0.058
eGFR, mL/min/1.73 m^2^	45 (19)	0.13 (0.05 to 0.21)	0.001
Creatinine clearance, mL/min	66 (26)	0.13 (0.05 to 0.20)	0.001
Urea, mmol/L ^#^	9.5 [7.2, 13.3]	−0.10 (−0.18 to −0.03)	0.007
**Markers of tubular damage**			
Urinary protein excretion, g/24 h ^#^	0.2 [0.0 to 0.5]	−0.01 (−0.09 to 0.06)	0.7
Urinary liver-type fatty acid-binding protein, µg/24 h ^#^	2.06 [0.91 to 7.04]	−0.06 (−0.14 to 0.03)	0.2
Urinary endothelial growth factor/creatinine ratio, ng/mg ^#^	6.5 [4.1 to 10.8]	0.08 (−0.00 to 0.16)	0.053
Plasma neutrophil-gelatinase associated lipocalin, µg/L ^#^	170 [133 to 232]	−0.07 (−0.15 to 0.00)	0.065
**Other heavy metals in plasma**			
Cadmium, µg/L	0.06 [0.04 to 0.07]	−0.14 (−0.21 to −0.06)	<0.001
Lead, µg/L	0.31 [0.22 to 0.44]	0.02 (−0.06 to 0.09)	0.6
Arsenic, µg/L	1.26 [1.04 to 2.04]	0.01 (−0.07 to 0.08)	0.8
Mercury, µg/L	0.29 [0.14 to 0.63]	0.07 (−0.01 to 0.14)	0.088
**Medication**			
Antihypertensive drugs, *n* (%)	592 (88.1)	−0.10 (−0.34 to 0.13)	0.4
Prednisolone, *n* (%)	665 (99.0)	−0.14 (−0.89 to 0.60)	0.7
Calcineurin inhibitor, *n* (%)	384 (57.1)	−0.07 (−0.22 to 0.09)	0.4
Proliferation inhibitor, *n* (%)	560 (83.3)	0.01 (−0.19 to 0.22)	0.9
mTOR inhibitor, *n* (%)	23 (3.4)	0.13 (−0.29 to 0.55)	0.5

Normally distributed data are presented as the mean ± standard deviation, skewed data as the median [interquartile range], and categorical data as the count (valid percentage). ^#^ Variables were log_2_-transformed to fulfill the assumptions of the linear regression analyses. Diabetes was defined according to the American Diabetes Association criteria [21]. Data on smoking status were missing for 40 patients (6.0%); data on donor age were missing for 19 patients (2.8%); data on HbA1c were missing for 26 patients (3.9%); data on hs-CRP were missing for 39 patients (5.8%); data on urinary endothelial growth factor were missing for 52 patients (7.7%); data on urinary liver-type fatty acid-binding protein were missing for 69 patients (10.1%). All other variables had missing data for ≤10 patients. Abbreviations: eGFR, estimated glomerular filtration rate as calculated using the creatinine and cystatin C-based CKD-EPI formula; hs-CRP, high-sensitivity C-reactive protein; mTOR, mammalian target of rapamycin.

**Table 2:** Univariable and adjusted Cox regression analyses of the associations of plasma thallium with graft failure and mortality.

	Graft Failure		All-Cause Mortality	
Model	HR per SD (95% CI)	*p*-Value	HR per SD (95% CI)	*p*-Value
Crude	0.89 (0.72 to 1.10)	0.3	0.87 (0.74 to 1.01)	0.073
Model 1	0.90 (0.72 to 1.11)	0.6	1.02 (0.86 to 1.20)	0.8
Model 2	0.90 (0.72 to 1.11)	0.3	1.00 (0.85 to 1.19)	0.9
Model 3	0.90 (0.72 to 1.13)	0.4	1.02 (0.87 to 1.21)	0.8

In total, 80 patients (11.9%) developed graft failure, and 143 (21.3%) died during the median follow-up of 5.4 [IQR: 4.8 to 6.1] years. Model 1 was adjusted for age, sex, estimated glomerular filtration rate as calculated using the creatinine- and cystatin C-based CKD-EPI formula, and log_2_ 24 h urinary protein excretion. Model 2 was adjusted for the variables in Model 1 plus pre-emptive transplantation and history of cardiovascular disease. Model 3 was adjusted for the variables in model 2 plus log_2_ plasma cadmium, log_2_ plasma arsenic, log_2_ plasma lead, and log_2_ plasma mercury. CI, confidence interval; HR, hazard ratio; SD, standard deviation.

## Data Availability

Data is available upon reasonable request to the corresponding author.

## Supplementary Materials

The following are available online at https://www.mdpi.com/article/10.3390/jcm11071970/s1. Figure S1: Diagram visualizing the flow of participants through the study. Figure S2: Kaplan–Meier analyses for death-censored graft survival and patient survival for patients with plasma thallium concentrations >90th percentile. The *p*-value represents the significance of difference between the groups as assessed using a log-rank test. Figure S3: Kaplan–Meier analyses for death-censored graft survival and patient survival for patients with plasma thallium concentrations >95th percentile. The *p*-value represents the significance of difference between the groups as assessed using a log-rank test. Table S1: Cox regression analyses with transformed plasma thallium concentrations to identify potential non-linear associations with outcome.
