# Supplementary material for: Plasma Thallium Concentration, Kidney Function, Nephrotoxicity and Graft Failure in Kidney Transplant Recipients
Source: J Clin Med. 2022 Apr 1;11(7):1970. doi: 10.3390/jcm11071970 (PMC9000150; doi:10.3390/jcm11071970)
Supplement: Supplementary file 1 [file jcm-11-01970-s001.zip › supplementary materials .pdf]

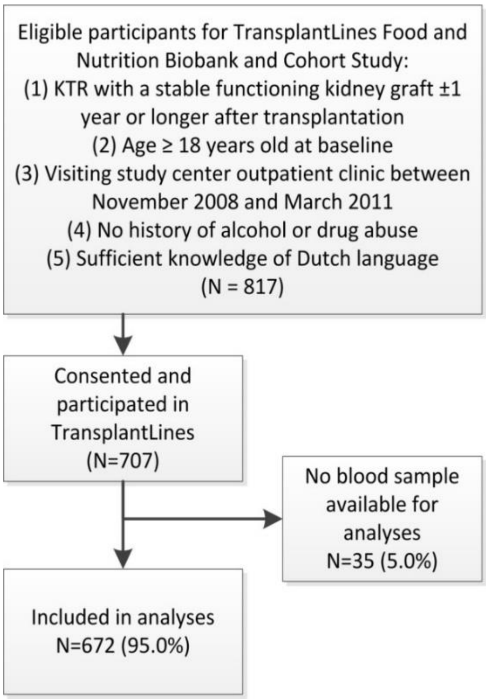

Figure S1. Diagram visualizing the flow of participants through the study.

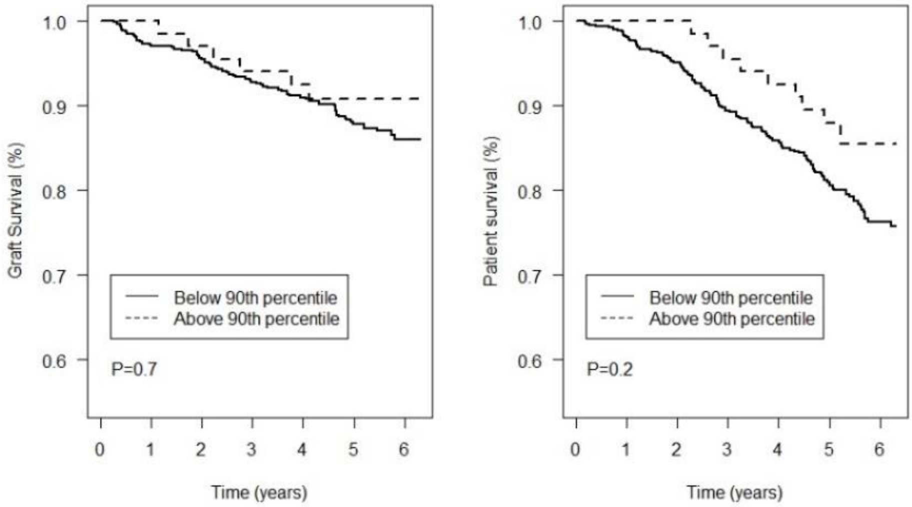

Figure S2 Kaplan-Meier analyses for death-censored graft survival and patient survival for patients with plasma thallium concentrations >90th percentile. P-value represents significance of difference between the groups as assessed using log-rank test.

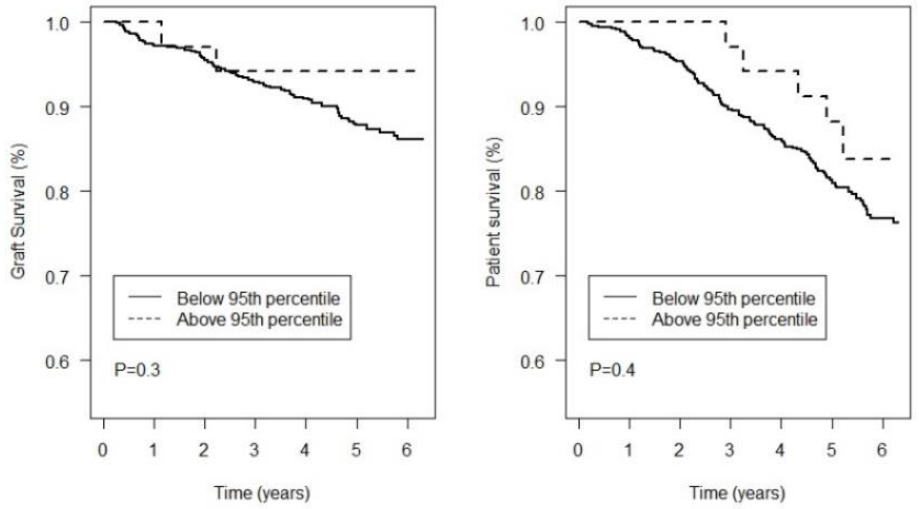

Figure S3. Kaplan-Meier analyses for death-censored graft survival and patient survival for patients with plasma thallium concentrations >95th percentile. P-value represents significance of difference between the groups as assessed using log-rank test.

Table S1. Sensitivity analyses: Cox regression analysis of the associations of plasma thallium concentrations with graft failure and mortality using various transformations in adjusted Cox regression models.

	**Graft Failure (n = 80; 11.9%)**		**Mortality (n = 143; 21.3%)**	
**Transformation**	**HR per SD (95% CI)**	**P-value**	**HR per SD (95% CI)**	**P-value**
Log_2 (plasma thallium)	0.91 (0.70 to 1.19)	0.5	1.08 (0.88 to 1.32)	0.5
Square root (plasma thallium)	0.26 (0.02 to 3.81)	0.3	1.93 (0.26 to 14.27)	0.5
1 / (plasma thallium)	1.00 (0.97 to 1.02)	0.8	0.99 (0.98 to 1.01)	0.6
(plasma thallium)^2	*Not interpretable*#	0.2	*Not interpretable*#	0.6

All presented values concern the association of plasma thallium concentrations with outcomes in models adjusted for age, sex, estimated glomerular filtration rate as calculated using the creatinine and cystatin C-based CKD-EPI formula, log_2 24h urinary protein excretion pre-emptive transplantation, history of cardiovascular disease. # Values not presented in table to avoid confusion; because of the transformation the point estimates and 95% CI may seem confusing. Values are as follows: HR 0.00 (0.00 to 758.94) for graft failure, and HR 20.86 (0.00 to 954214.30).
